# The fate of indeterminate liver lesions: What proportion are precursors of hepatocellular carcinoma?

**DOI:** 10.1186/s12876-022-02135-x

**Published:** 2022-03-10

**Authors:** Sara Cococcia, Priti Dutta, Melika Moghim, Brian Hogan, Sudeep Tanwar, Aileen Marshall, Douglas Macdonald, Dominic Yu, James O’Beirne, William M. Rosenberg, Paul M. Trembling

**Affiliations:** 1 Sheila Sherlock Liver Centre, Royal Free London NHS Foundation Trust, Pond Street, London, NW3 2QG UK; 2 Institute for Liver and Digestive Health, Division of Medicine, University College London, Royal Free Hospital, Rowland Hill Street, London, NW3 2PF UK; 3 First Department of Internal Medicine, San Matteo Hospital Foundation, University of Pavia, Pavia, Italy; 4 Department of Radiology, Royal Free London NHS Foundation Trust, Pond Street, London, NW3 2QG UK; 5 Department of Hepatology, Sunshine Coast University Hospital, Birtinya, QLD Australia

**Keywords:** Hepatocellular carcinoma, Liver cirrhosis, Liver neoplasms, Magnetic resonance imaging

## Abstract

**Background:**

The natural history and incidence of hepatocellular carcinoma (HCC) arising from indeterminate liver lesions are not well described. We aimed to define the incidence of HCC in a cohort of patients undergoing surveillance by magnetic resonance imaging (MRI) and estimate any associations with incident HCC.

**Methods:**

We performed a retrospective follow-up study, identifying MRI scans in which indeterminate lesions had been reported between January 2006 and January 2017. Subsequent MRI scan reports were reviewed for incident HCC arising from indeterminate lesions, data were extracted from electronic patient records and survival analysis performed to estimate associations with baseline factors.

**Results:**

One hundred and nine patients with indeterminate lesions on MRI were identified. HCC developed in 19 (17%) patients over mean follow up of 4.6 years. Univariate Cox proportional hazards analysis found incident HCC to be significantly associated with baseline low platelet count (hazard ratio (HR) = 7.3 (95% confidence intervals (CI) 2.1–24.9), high serum alpha-fetoprotein level (HR = 2.7 (95% CI 1.0–7.1)) and alcohol consumption above fourteen units weekly (HR = 3.1 (95% CI 1.1–8.7)). Multivariate analysis, however, found that only low platelet count was independently associated with HCC (HR = 5.5 (95% CI 0.6–5.1)).

**Conclusions:**

HCC arises in approximately one fifth of indeterminate liver lesions over 4.6 years and is associated with a low platelet count at the time of first diagnosis of an indeterminate lesion. Incidence of HCC was more common in people with viral hepatitis and in those consuming > 14 units of alcohol per week. Our data may be used to support a strategy of enhanced surveillance in patients with indeterminate lesions.

**Supplementary Information:**

The online version contains supplementary material available at 10.1186/s12876-022-02135-x.

## Background

Liver cancer incidence is increasing worldwide, now representing the fifth commonest cancer and the second most frequent cause of cancer-related death [1]. Hepatocellular carcinoma (HCC) accounts for 90% of primary liver cancers [1] and 80–90% of HCCs occur in individuals with underlying cirrhosis [2], with viral hepatitis B and C infection and alcohol related liver disease (ArLD) being the most common aetiologies [3]. A number of studies have demonstrated that the incidence of HCC in cirrhotic patients is around 1.5% per year or greater, regardless of the underlying chronic liver disease (CLD) aetiology [4–6]. Therefore, both European and American guidelines now recommend six-monthly screening ultrasound scans (USS) to be performed in all cirrhotic patients in order to facilitate early HCC diagnosis [1, 7]. When a suspicious lesion is revealed by the screening USS, a second level of imaging, either a computed tomography (CT) or a magnetic resonance imaging (MRI) scan, should be carried out. Although non-invasive criteria for the diagnosis of HCC have been formulated for both these techniques, MRI has been found to have a higher specificity and sensitivity when compared to CT, particularly when it comes to small lesions [1, 8, 9], and there have been advancements in diagnostic accuracy using MRI imaging, for example with the use of hepatospecific contrast media [10]. Lesions, however, do not always demonstrate the characteristic radiological features of HCC (arterial enhancement, and venous phase washout and / or delayed phase washout) [1, 11], or of any other known liver lesions, and such lesions are therefore classified as indeterminate. These indeterminate lesions are commonly seen in clinical practice, usually in cirrhotic patients, posing a diagnostic and prognostic dilemma for clinicians. Whilst guidelines are more explicit regarding management of lesions of 1 cm or more in size, including consideration of biopsy, optimal management of smaller lesions remains unclear.

There is a scarcity of data describing the natural history or malignant potential of such indeterminate lesions, with a reported HCC incidence that varies from 14 to 56% [12, 13]. A recent study investigating the incidence of HCC after directly acting antiviral (DAA) therapy for chronic hepatitis C (CHC) virus infection, showed that the relative risk for developing HCC was 2.93 for those with an indeterminate lesion at surveillance USS pre-therapy [14]. Risk factors predicting progression to malignancy are still unclear. Diagnostic algorithms are needed to identify the patients at higher risk of progression and in order to plan the provision of resources to permit surveillance with MRI scanning rather than USS for the large number of indeterminate liver lesions encountered.

In light of the lack of data on the natural history of indeterminate liver lesions, we aimed to determine the risk of transformation of indeterminate lesions to HCC after a diagnosis of such a lesion on an index MRI scan and to evaluate potential predictive factors of progression. We also investigated the clinical utility of the Liver Imaging Reporting and Data System (LI-RADS) in predicting HCC in our cohort.

## Methods

### Patient population and study design

We performed a retrospective follow-up study at the Royal Free London NHS Foundation Trust, which manages 1.6 million patients each year and provides secondary and tertiary care for patients with CLD. The electronic radiology records of our hospital were interrogated to identify all patients who had undergone an MRI scan showing an indeterminate lesion between January 2006 and January 2017. It is usual practice in our unit, following the identification of an indeterminate lesion, and after review by the unit’s multidisciplinary team, to follow up with sequential MRI scans. The diagnosis of a lesion as indeterminate is based on absence of typical radiological hallmarks of HCC [1] and lacks a distinct definition based on positive radiological findings that could form the basis for comparison with the published literature. Previous studies have included participants by searching reports for lesions “without the full complement of characteristics of HCC” [15], or “not demonstrating enhancement greater than the liver in the arterial phase, and less than the liver on the venous or delayed phases” [12]. Our search terms included all terms recorded in MRI reports of lesions that do not fulfil the radiological diagnosis of HCC but trigger entry in to an MRI surveillance programme in our unit. To identify relevant scans, the search was conducted using the following keywords: “indeterminate”, “regenerative”, “nodule”, “arterialised” and “dysplastic”. Index reports were reviewed to identify eligible patients, who were included if they were aged 18 years or more and had at least two years follow-up from the index scan, unless an HCC occurred in the first two years. Exclusion criteria included a history of HCC or an HCC revealed by the index MRI, or an alternative diagnosis of the liver lesion made through further investigations. Characteristics of the index lesion, including size, number of lesions and description (e.g. “indeterminate”, “regenerative” etc.) were retrieved from all scan reports. Demographic data and relevant clinical information (age, sex, ethnicity, liver function tests, CLD aetiology, smoking status, alcohol intake, concomitant or previous malignancies and family history of HCC) were collected from clinical records. If available, serum alfa-fetoprotein (AFP) values at every check point, and histological reports were recorded.

Date of diagnosis of indeterminate lesions was defined as the date of the first MRI scan reporting the studied lesion. Follow-up scans were reviewed to identify transformation of indeterminate lesions to HCC. We defined a diagnosis of HCC if the HCC occurred in the position of a previously identified indeterminate lesion. HCC was diagnosed based on one or more of radiological criteria, biochemical parameters, histological assessment or consensus opinion as part of the patient’s usual clinical management. All MRI scans were reported by experienced radiologists working in collaboration with the liver unit of our institution.

The Liver Imaging Reporting and Data System (LI-RADS) is a classification system for liver lesions in patients with CLD (and in patients with chronic hepatitis B (CHB) without cirrhosis), comprising a score ascribed to each lesion corresponding to degree of suspicion for HCC, the highest score (LI-RADS 5) classified as ‘definitely HCC’ [16]. In order to evaluate performance of the LI-RADS system in predicting HCC in our population the index MRI scans were rescored according to LI-RADS classification (version 2018) by an experienced liver radiologist (PD). As per guidance for LI-RADS scoring, a score was not ascribed in patients with CLD due to rare causes (e.g. vascular disease, common variable immune deficiency, Noonan syndrome or situs inversus viscerum).

Scans were performed on a Philips Achieva 1.5 Tesla MRI scanner and a Philips Ingenia 1.5 Tesla MRI scanner. Dotarem® (gadoterate meglumine), a gadolinium-based contrast agent, was used to image the patients in this study.

### Statistical analysis

According to their distribution, continuous data were described with either mean and standard deviation (SD) or median and interquartile range (IQR), whilst categorical data were described as counts and proportions.

The primary endpoint was first radiological diagnosis of HCC at the position of a previous indeterminate lesion on any subsequent MRI scan. Patients contributed person-years until the date of first presentation with transformation to HCC or censoring (last recorded scan with no HCC in those with no HCC). Survival time was calculated as the interval between first MRI scan showing an indeterminate lesion and first MRI scan to diagnose HCC (cases), or date of most recent MRI scan for patients with no HCC during follow up. Overall survival and HCC-free survival stratified by potential clinical and biochemical risk factors were summarised by Kaplan Meier curves, and any significant baseline covariates determined using the Log Rank test. To further explore the influence of clinical and biochemical parameters on HCC incidence, Cox proportional hazards models were generated. Univariate models were used to estimate association between each covariate and HCC by calculating hazard ratios (HR). Alanine aminotransferase (ALT), AFP, platelet count and albumin level were analysed both as continuous variables and as categorical variables using the upper or lower limit of the normal range as the cut-off. Any covariates demonstrating a significant association with HCC incidence at the site of a previous indeterminate lesion were entered into a multivariate model to estimate independent association.

Laboratory reporting thresholds were used to define upper and lower limits of normal for biochemical parameters. Continuous covariates were tested for normal distribution and those showing a skewed distribution were transformed using the natural logarithm prior to analysis. A two‐sided *p* < 0.05 was considered statistically significant in all analyses. Statistical analyses were performed using SPSS (Version 26.0. Armonk, NY: IBM Corp).

## Results

We identified one hundred and nine patients in whom indeterminate lesions had been identified through MRI scans. HCC developed from an indeterminate lesion in nineteen patients (17%) over a mean follow-up of 4.6 years (SD 2.7 years). One patient developed a metachronous *de-novo* HCC alongside an indeterminate lesion and was considered a censored case in our analysis, censored at the date of diagnosis of the *de-novo* HCC as no further surveillance imaging was performed. Median interval from index MRI to transformation to HCC was 493 days (IQR 872 days). In those with no HCC arising from indeterminate lesions, median follow up was 1690 days (IQR 1265 days). Total person-years of follow up was 503.6, equivalent to an event rate of 38 HCCs per 1000 patient years.

Twelve cases of HCC transformation (70.6%) were diagnosed within two years of follow up. All HCCs were diagnosed within the first four scans subsequent to the index imaging. The median intervals between the index scan and each subsequent scan were 1.4, 2.6, 3.7, 4.6, 5.6, 6.4, 7.3, 8.8, 9.7 and 10.7 years.

Baseline patient characteristics are shown in Table 1. The mean age of patients at the time of the index scan was 52.4 (± 14.5) years with approximately the same proportion of male and female (54.1% vs. 45.9%). In the whole study cohort, white ethnicity was the most represented (58.7%), followed by black ethnicity (17.4%). However, among those who developed HCC in indeterminate lesions, the second most represented ethnicity was Asian (10.5%). The majority of patients were non-smokers (64.2%) and had an underlying diagnosis of cirrhosis (80.7%). The most common cirrhosis aetiology was CHC (35.8%) followed by CHB (12.8%) and ArLD (9.2%). Only three patients had a family history of HCC, none of these developed HCC. Four patients had a history of non-HCC malignancies, and none of these developed HCC. Baseline demographic and clinical characteristics were similar when comparing those who did and those who did not develop HCC, with the exception of platelet count which was significantly lower among those who progressed to HCC (*p* < 0.001). None of the non-cirrhotic patients developed HCC during the follow up period.

**Table 1:** Demographic and clinical characteristics of included patients

	Patients who developed HCC (n = 19)	Patients who did not develop HCC (n = 90)	All patients (n = 109)	*P* value
Age (mean, SD)	54.2 (± 10.0)	51.8 (± 15.1)	52.4 (± 14.5)	0.398
Sex; n (%)				0.385
Male	12 (63.2%)	47 (52.2%)	59 (54.1%)	
Female	7 (36.8%)	43 (47.8%)	50 (45.9%)	
BMI (mean, SD)	27.6 (± 3.9)	27.7 (± 5.5)	27.7 (± 5.3)	0.990
Ethnicity; n (%)				
White	15 (78.9%)	49 (54.4%)	64 (58.7%)	
Black	1 (5.3%)	18 (20%)	19 (17.4%)	
Asian	2 (10.5%)	11 (12.2%)	13 (11.9%)	
Other	1 (5.3%)	10 (11.1%)	11 (10.1%)	
Mixed	0 (0%)	1 (1.1%)	1 (0.9%)	
Missing	0 (0%)	1 (1.1%)	1 (0.9%)	
Smoking status; n (%)				
Non-smoker	9 (47.4%)	61 (67.8%)	70 (64.2%)	
Smoker	7 (36.8%)	19 (21.1%)	26 (23.9%)	
Ex-smoker	3 (15.8%)	8 (8.9%)	11 (10.1%)	
Missing	0 (0%)	2 (2.2%)	2 (1.8%)	
Main aetiology; n (%)				
HCV	10 (52.6%)	29 (32.2%)	39 (35.8%)	
HBV	2 (10.5%)	12 (13.3%)	14 (12.8%)	
HBV/HDV	0 (0%)	6 (6.7%)	6 (5.5%)	
ArLD	4 (21.1%)	6 (6.7%)	10 (9.2%)	
NAFLD/NASH	0 (0%)	6 (6.7%)	6 (5.5%)	
Autoimmune	1 (5.3%)	2 (2.2%)	3 (2.8%)	
Other	2 (10.5%)	29 (32.2%)	31 (28.4%)	
Alcohol consumption				0.023
≤ 14 U/week	94 (86.2%)	80 (88.9%)	14 (73.7%)	
> 14 U/week	12 (11%)	7 (7.8%)	5 (26.3%)	
Missing	3 (2.8%)	3 (3.3%)	0	
Cirrhosis; n (%)	19 (100%)	69 (76.7%)	88 (80.7%)	0.019
Other malignancy; n (%)	0 (0%)	4 (4.4%)	4 (3.7%)	0.346
Family history of HCC; n (%)	0 (0%)	3 (3.5%)	3 (2.9%)	0.409
ALT (unit/l)	49 (IQR 46.5)	45 (IQR 67.3)	45 (IQR 61)	0.531
AST (unit/l)	52 (IQR 22.5)	51 (IQR 58.3)	51 (IQR 53)	0.227
ALP (unit/l)	101 (IQR 36)	96 (IQR 65.3)	96 (IQR 63)	0.333
Albumin (gram/l)	40.3 (± 4.5)	43.2 (± 4.7)	42.6 (± 4.5)	0.014
Platelet count (× 10^9^/l)	90 (IQR 77)	157 (IQR 89.8)	148 (IQR 95.5)	< 0.001
AFP (kunits/l)	8.9 (IQR 17)	4 (IQR 5)	4.4 (IQR 7)	0.074
Description of index lesion(s); n(%)				
Arterialised	15 (78.9)	63 (70.0)	78 (71.6)	0.432
Dysplastic	1 (5.3)	1 (1.1)	2 (1.8)	0.220
Indeterminate	15 (78.9)	77 (85.6)	92 (84.4)	0.471
Regenerative	4 (21.1)	22 (24.4)	26 (23.9)	0.753
Perfusional changes	0 (0)	7 (7.8)	7 (6.4)	0.209

AFP, alphafetoprotein; ArLD, alcohol-related liver disease; ALP, alkaline phosphatase; ALT, alanine aminotransferase; AST, aspartate aminotransferase; BMI, body mass index; HBV, hepatitis B virus; HCC, hepatocellular carcinoma; HCV, hepatitis C virus; HDV, hepatitis delta virus; NAFLD, non-alcoholic fatty liver disease; NASH, non-alcoholic steatohepatitis

The radiological descriptions of the index lesion(s) were similar between the two groups.

Data on size of largest lesion on index scan was available in 93 patients. HCC was found in 10 (20%) of 49 patients where the largest lesion was ≥ 1 cm compared to 7 patients (16%) of 44 patients with a largest lesion of < 1 cm, although there was not a significant association between HCC and lesion size of ≥ 1 cm on univariate regression analysis (Table 2). In contrast, in the 20 patients where the largest lesion was ≥ 2 cm, HCC occurred in 3 (15%), compared to 14 (19%) of the 73 patients with a lesion < 2 cm in diameter. Again, this association was not significant on univariate regression analysis. The most common aetiology in those who developed HCC was CHC (53%), followed by ArLD (21%) and CHB (11%). There were no cases in which the underlying aetiology was non-alcoholic fatty liver disease (NAFLD). Twenty six percent of patients with CHC, 14% with CHB and 40% with ArLD developed HCC, however these rates were not significantly different between groups. In the cohort that did not develop HCC from indeterminate lesions during follow up, CHC was also the most common aetiology (32%), followed by CHB in 13%, ArLD in 7%, NAFLD in 7% and hepatitis B and D virus co-infection in 7%. Although only two patients had a recorded diagnosis of CLD due to both CHC and alcohol (one of whom developed HCC), of the six patients with a diagnosis of CHC reporting alcohol use of more than 14 units / week, four developed HCC. Of the four patients with a diagnosis of CHC consuming > 21 units / week, two developed HCC.

**Table 2:** Univariate Cox regression analysis

Covariate	Continuous/Categorical		Hazard ratio	95% CI	*P* value	Data available (N)
Sex	Categorical	Female	Reference			109
		Male	1.444	0.568–3.667	0.440	
Age at diagnosis	Continuous		1.015	0.982–1.050	0.377	109
BMI	Continuous		0.994	0.913–1.082	0.889	92
	Categorical	Normal BMI	Reference			
		Overweight or obese	1.062	0.403–2.796	0.903	
Smoking status	Categorical	Non-smoker	Reference			107
		Smoker or ex-smoker	2.308	0.936–5.694	0.069	
		Non-smoker or ex-smoker	Reference			
		Smoker	2.027	0.796–5.161	0.138	
Alcohol use	Categorical	14 units or less per week	Reference			106
		More than 14 units per week	3.117	1.121–8.672	**0.029**	
Platelet count	Continuous		0.982	0.972–0.992	< 0.001	106
	Categorical	Normal range	Reference			
		Below LLN	7.259	2.114–24.920	**0.002**	
ALT*	Continuous		1.099	0.646–1.872	0.728	106
	Categorical	Normal range	Reference			
		Above ULN	1.291	0.508–3.280	0.591	
AST*	Continuous		1.460	0.734–2.904	0.281	106
	Categorical	Normal range	Reference			
		Above ULN	1.747	0.702–4.343	0.230	
ALP*	Continuous		1.406	0.518–3.816	0.504	106
	Categorical	Normal range	Reference			
		Above ULN	1.406	00.518–3.816	0.504	
Albumin	Continuous		0.914	0.852–0.981	0.013	106
	Categorical	Normal range	Reference			
		Below LLN	1.632	0.217–12.266	0.634	
AFP*	Continuous		1.433	0.958–2.144	0.080	97
	Categorical	Normal range	Reference			
		Above ULN	2.715	1.042–7.074	**0.041**	
Largest index lesion diameter	Categorical	Less than 1 cm versus 1 cm or more	Reference			93
			1.296	0.493–3.406	0.599	
		Less than 2 cm versus 2 cm or more	Reference			
			0.790	0.227–2.753	0.712	

Estimates of association with HCC are presented as hazard ratios for each covariate

CI, confidence intervals; BMI, body mass index; LLN, lower limit of normal; ALT, alanine aminotransferase; ULN, upper limit of normal; AST, aspartate aminotransferase; ALP, alkaline phosphatase; AFP, alphafetoprotein

*Data were positively skewed, therefore transformed by the natural logarithm before regression analysis as a continuous variable

*P* values in bold represent statistically significant values at the 0.05 level

Kaplan–Meier survival analysis showed significant survival differences between alcohol groups (those reporting consumption of 14 units or less of alcohol weekly and those consuming above this threshold), baseline platelet count (above and below the lower limit of normal) and baseline serum AFP groups (above and below the upper limit of normal) (Table 3 and Additional file 1: Supplementary material).

**Table 3:** Log rank test results from Kaplan–Meier estimates of survival

Covariate		Log rank	*P* value
BMI	Normal BMI versus overweight or obese	0.015	0.903
Smoking status	Non-smoker versus smoker or ex-smoker	3.489	0.062
	Non-smoker or ex-smoker versus smoker	2.289	0.130
Alcohol use	14 units or less versus more than 14 units per week	5.275	**0.022**
Platelet count	Normal range versus below LLN	13.618	** < 0.001**
ALT	Normal range versus above ULN	0.290	0.590
AST	Normal range versus above ULN	1.477	0.224
ALP	Normal range versus above ULN	0.001	0.976
Albumin	Normal range versus below LLN	0.231	0.631
AFP	Normal range versus above ULN	4.532	**0.033**
Largest index lesion diameter	Less than 1 cm versus 1 cm or more	0.278	0.598
	Less than 2 cm versus 2 cm or more	0.137	0.711

*P* values in bold represent statistically significant values at the 0.05 level

BMI, body mass index; LLN, lower limit of normal; ALT, alanine aminotransferase; ULN, upper limit of normal; AST, aspartate aminotransferase; ALP, alkaline phosphatase; AFP, alphafetoprotein

Univariate Cox regression analysis showed a significant association between incidence of HCC transformation and alcohol consumption of more than 14 units weekly (HR = 3.117 (95% confidence intervals (CI) 1.121–8.672)), baseline platelet count below the lower limit of normal (HR = 7.259 (95% CI 2.114–24.920)), and baseline serum AFP level above the upper limit of normal (HR = 2.715 (95% CI 1.042–7.074)) (Table 2).

Multivariate Cox analysis comprising the variables with significant association in univariate analyses showed that a significant independent association remained with a low platelet count (HR = 5.535 (95% CI 1.550–19.759)) (Table 4).

**Table 4:** Multivariate Cox regression analysis

Covariate		Hazard ratio	95% CI	*P* value	Data available (N)
Alcohol use	14 units or less	Reference			97
	More than 14 units	1.767	0.609–5.128	0.295	
Platelet count	Normal range	Reference			
	Below LLN	5.535	1.550–19.759	**0.008**	
AFP	Normal range	Reference			
	Above ULN	2.549	0.978–6.643	0.056	

Estimates of association with HCC are presented as hazard ratios for each covariate

*P* values in bold represent statistically significant values at the 0.05 level

CI, confidence intervals; LLN, lower limit of normal; AFP, alphafetoprotein; ULN, upper limit of normal

It was possible to calculate the LI-RADS score in 93 of the 109 patients. The majority of the lesions (71%) were classified as LI-RADS 3, followed by LI-RADS 2 (21.5%) and 4 (6.5%). One patient was classified as LI-RADS 1. The percentages of subsequent HCCs according to baseline LI-RADS scores of 1, 2, 3 and 4 were 0, 15, 19.7 and 33.3%, respectively.

## Discussion

We have shown that over an average of nearly five years of follow-up, almost a fifth of patients found to have an indeterminate liver lesion on MRI developed HCC from the index indeterminate lesion. Incidence of HCC transformation at two years of follow-up was 11%. We found a significant association between low platelet count at the time of diagnosis of an indeterminate lesion and subsequent development of HCC from that indeterminate lesion. Incidence was noted to be particularly high in patients with CHC who consumed the most alcohol. The risk of HCC in individuals with CLD is well-recognised, and it is known that viral hepatitis confers an increased risk within this population [17]. Our data show that presence of indeterminate hepatic lesions substantially increases incidence of HCC above this background risk and so should be considered a pre-malignant condition. Data on initial lesion size was not conclusive, with more HCCs seen in those with lesion size ≥ 1 cm compared to < 1 cm, but fewer HCCs seen in those with lesion size ≥ 2 cm compared to < 2 cm. There were no significant associations between lesion size and HCC on regression analysis, and this question may be answered with larger studies. Our analysis of progression to HCC based on baseline LI-RADS score showed increasing incidence with increasing score, but our rates were lower than those recently reported in an American study [18]. Possible reasons for this include a higher mean age and the exclusion of some lesions considered not worrisome for HCC according to LI-RADS criteria in the American study. Further, the definition of “a lesion” employed in the American study was more restricted than in our study, only including participants where an ‘arterially enhancing lesion’ was reported by the radiologist.

There are few data describing the natural history of indeterminate hepatic lesions and the incidence of transformation to HCC. Indeed, the European Association for the Study of the Liver (EASL) call for “further efforts to adopt standardised and unique definitions worldwide for the diagnosis of HCC” [1]. A study based in the USA followed two hundred and fifty-two patients with at least one indeterminate lesion on imaging over four years and reported an incidence of HCC of 21% [15]. As in our study, CHC was the most prevalent aetiology, and low platelet count was an independent predictor of HCC. In a retrospective study of cirrhotic patients with indeterminate nodules of 1–2 cm diameter, HCC was diagnosed, either radiologically and / or histologically in 14% of eighty patients over a mean of two and a half years [12]. An Australian study of histopathological findings of biopsies of indeterminate lesions found HCC in 56% of specimens, further adding to the importance of following up lesions that do not meet radiological criteria for HCC [13]. A retrospective study of 127 indeterminate lesions of less than 2 cm in diameter in seventy-three patients, followed up with CT, showed that 16% of nodules became HCCs during two years of follow up [19]. A Spanish study followed up 1123 patients with cirrhosis due to hepatitis C virus, treated with DAA medication. An indeterminate lesion was reported on USS in 80 (7%) of the patients prior to starting therapy. Following therapy 13 (16.25%) of these individuals developed an HCC, however only 46% of the reported HCCs developed from the same indeterminate lesion previously described [14].

Strengths of this study include the relatively long follow period (compared to previous studies) and the access to linked demographic, biochemical and clinical data. We used radiological and, where clinically indicated and therefore available, histological data to determinate HCC diagnoses, which reflect the diagnostic decision-making process used in clinical practice. Limitations include the retrospective study design. Further, we were unable to access reports of MRI scans performed prior to 2006, limiting the follow up period. Demographic data including alcohol use and smoking status were taken from clinical records rather than direct questioning of patients. We aimed to maximise the identification of indeterminate lesions by using a range of key words, but some cases may have been missed due to the heterogenicity of terms used to describe indeterminate lesions.

Our study has added to the limited data available on the natural history of indeterminate lesions and reports similar incidence of HCC and associations to other studies. Our data supports a regimen of surveillance of indeterminate lesions and, although we found no incident cases of HCC beyond five and a half years, we propose that a prospective long-term outcome study would further inform guidance on surveillance and may provide further clarity on prognostic markers and could contribute to the development of a clinical tool to improve the identification of patients who may benefit from tailored surveillance of indeterminate lesions.

## Conclusions

The incidence of HCC arising from indeterminate lesions is high, occurring in nearly one fifth of patients followed over 5 years from the diagnosis of an indeterminate lesion. A low platelet count may be predictive of HCC in individuals with indeterminate lesions. The incidence of HCC was more common in people with viral hepatitis and in those consuming > 14 units of alcohol per week. Our study confirms observations of other groups and whilst supporting a strategy of enhanced surveillance in this population, indicates that further longitudinal data are needed to characterise the natural history of indeterminate lesions.

## Supplementary Information

**Additional file 1**. Kaplan-Meier survival curves showing HCC-free survival stratificed by presence or absence of potential clinical and biochemical risk factors. Significant covariates, according to log-rank testing, were alcohol use above 14 units/week, platelet count below lower limit of normal and AFP above upper limit of normal (Table 3).

## Data Availability

The datasets used and/or analysed during the current study are available from the corresponding author on reasonable request.

## Abbreviations

AFP: Alphafetoprotein
ArLD: Alcohol related liver disease
ALP: Alkaline phosphatase
ALT: Alanine aminotransferase
AST: Aspartate aminotransferase
BMI: Body mass index
CHB: Chronic hepatitis B
CHC: Chronic hepatitis C
DAA: Directly acting antiviral
HBV: Hepatitis B virus
HCC: Hepatocellular carcinoma
HCV: Hepatitis C virus
HDV: Hepatitis delta virus
LI-RADS: Liver Imaging Reporting and Data System
NAFLD: Non-alcoholic fatty liver disease
NASH: Non-alcoholic steatohepatitis
USS: Ultrasound scan
