# Supplementary material for: The fate of indeterminate liver lesions: What proportion are precursors of hepatocellular carcinoma?
Source: BMC Gastroenterol. 2022 Mar 10;22:118. doi: 10.1186/s12876-022-02135-x (PMC8908619; doi:10.1186/s12876-022-02135-x)
Supplement: Supplementary file 1 — Additional file 1. Kaplan-Meier survival curves showing HCC-free survival stratificed by presence or absence of potential clinical and biochemical risk factors. Significant covariates, according to log-rank testing, were alcohol use above 14 units/week, platelet count below lower limit of normal and AFP above upper limit of normal (Table 3). [file 12876_2022_2135_MOESM1_ESM.docx]

Supplementary material

Kaplan-Meier survival curves showing HCC-free survival stratificed by presence or absence of potential clinical and biochemical risk factors. Significant covariates, according to log-rank testing, were alcohol use above 14 units / week, platelet count below lower limit of normal and AFP above upper limit of normal (Table 3).

a) BMI


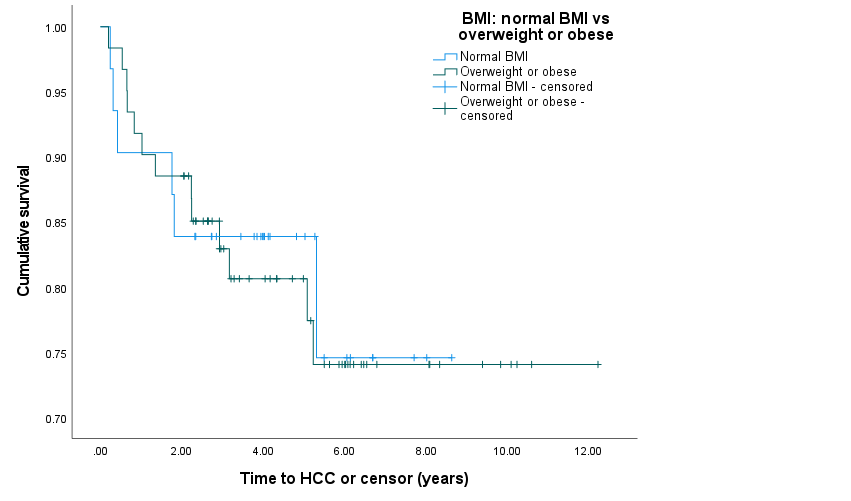


b) Smoking status: non-smoker vs smoker or ex-smoker


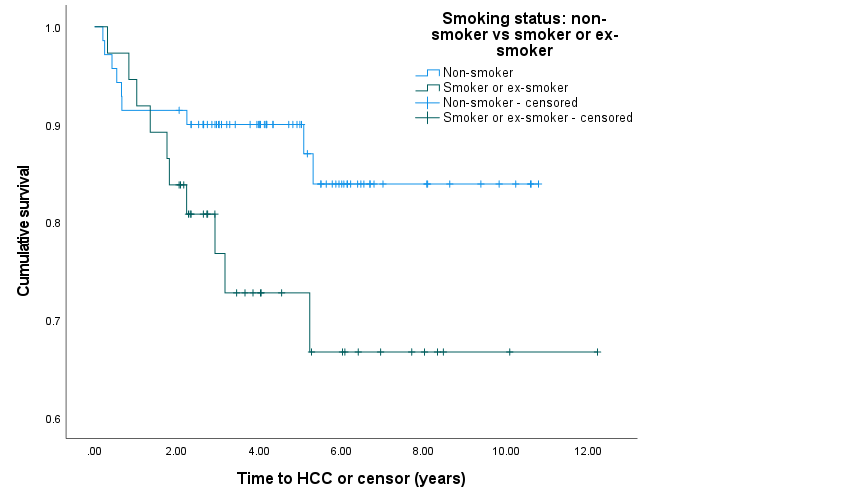


c) Smoking status: non-smoker or ex-smoker vs smoker


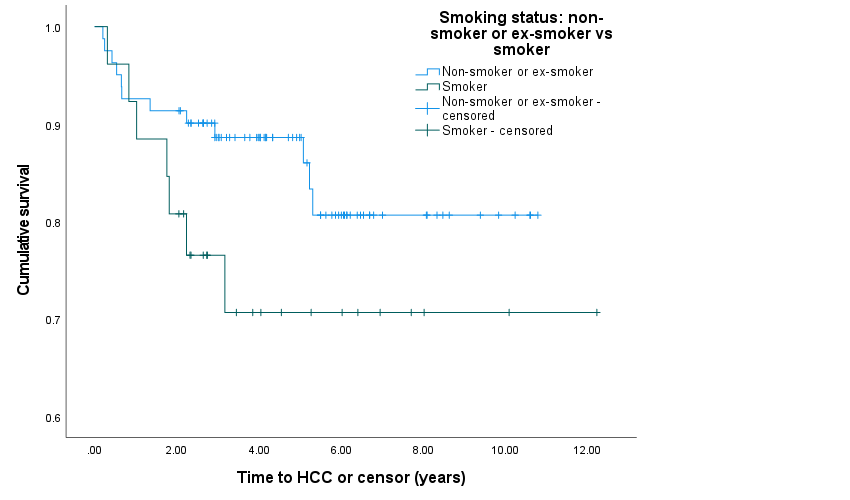


d) Alcohol use


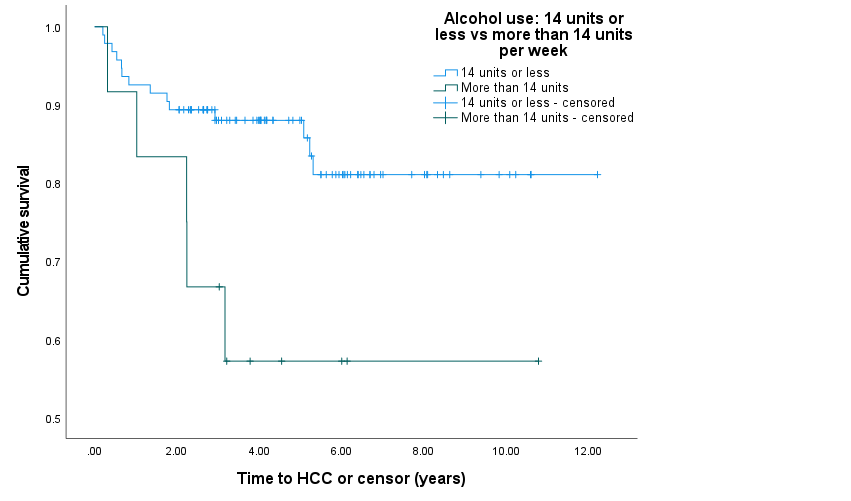


e) Platelet count


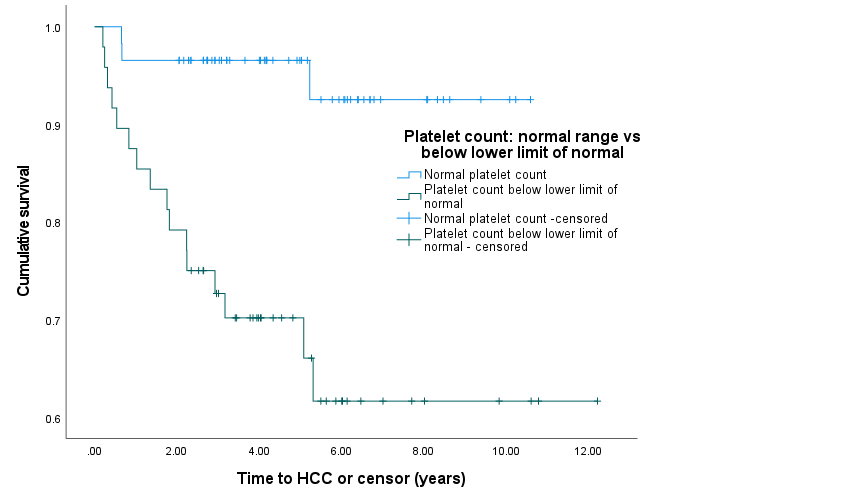


f) ALT


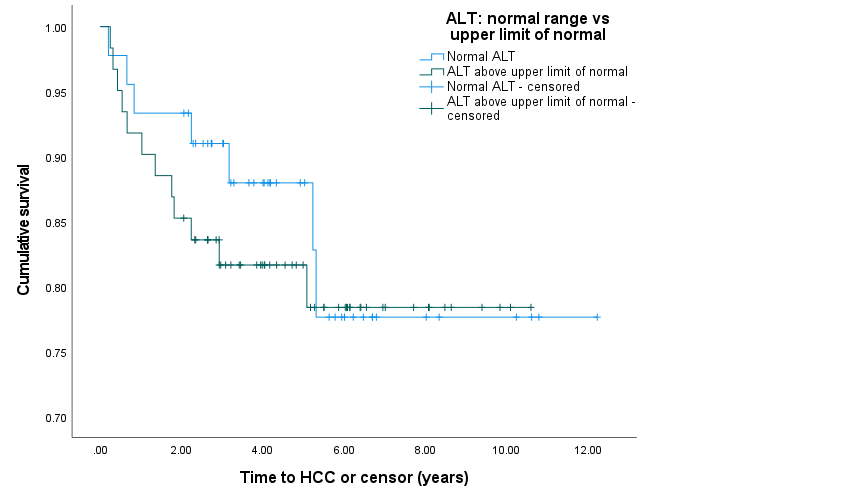


g) AST


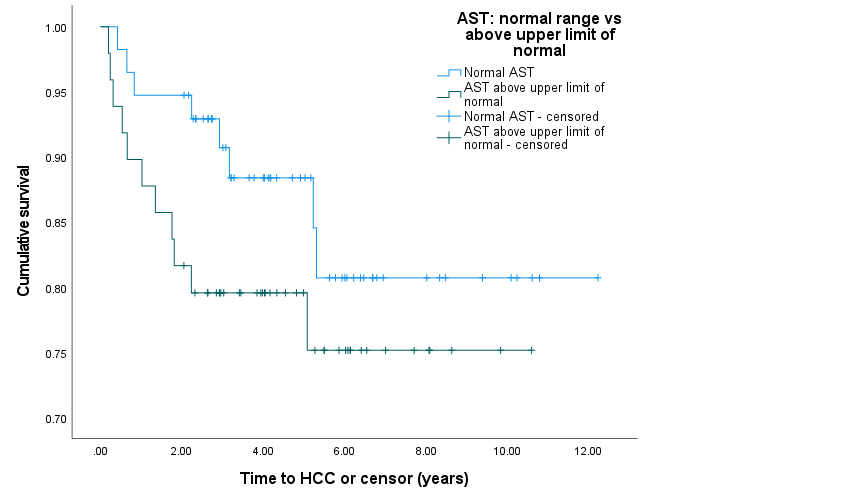


h) ALP


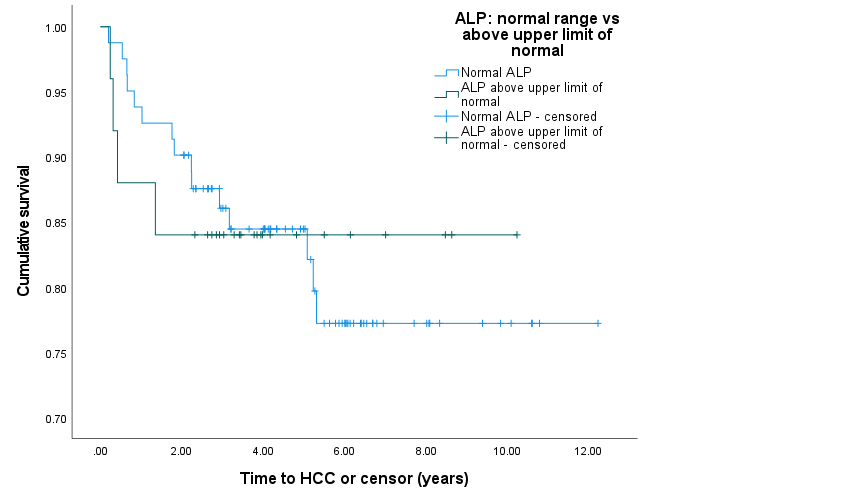


i) Albumin


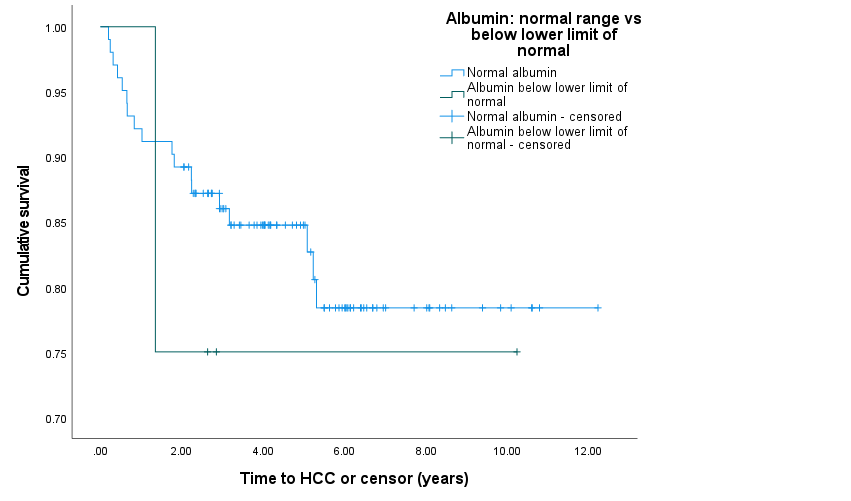


j) AFP


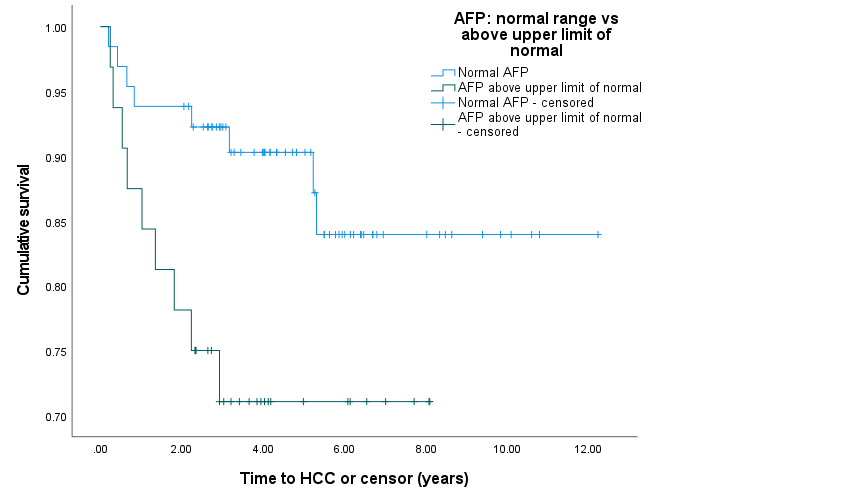


k) Index lesion less than 1 cm vs 1 cm or more


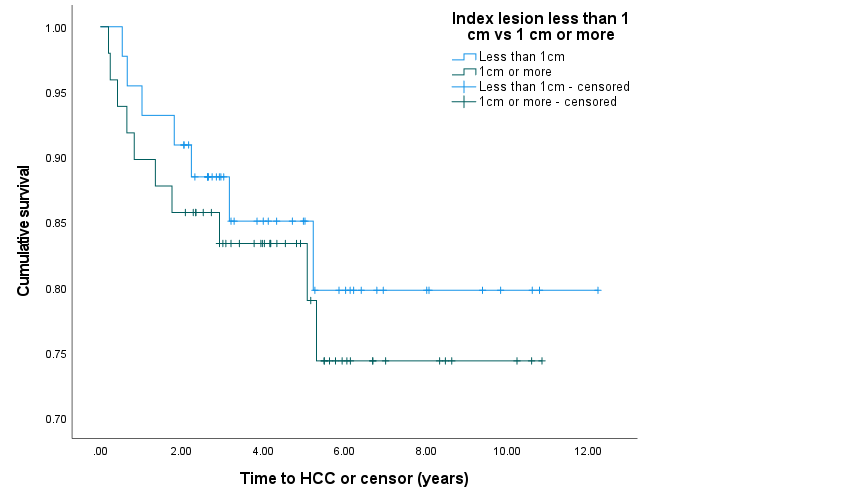


l) Index lesion less than 2 cm vs 2 cm or more


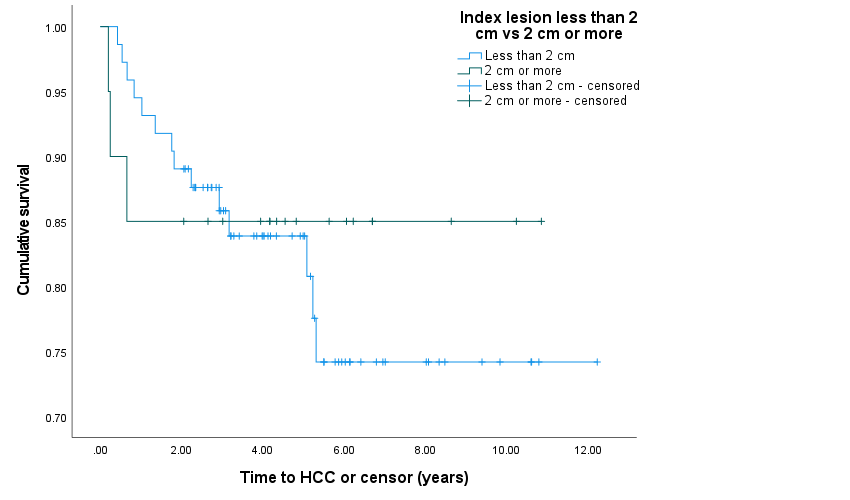


HCC, hepatocellular carcinoma; BMI, body mass index; ALT, alanine aminotransferase; AST, aspartate aminotransferase; ALP, alkaline phosphatase; AFP, alphafetoprotein
